# Improvement of Total Etching Dentin Bonding with Subpressure

**DOI:** 10.1038/s41598-017-07281-x

**Published:** 2017-07-28

**Authors:** Rui-Shen Zhuge, Yue-Ming Tian, Zu-Tai Zhang, Ning Ding, Yong-Mei Li, Dong-Xiang Zheng

**Affiliations:** 1 Beijing Institute of Dental Research, School of Stomatology, Capital Medical University, Beijing, 100050 China; 2 Department of Prosthodontics, School of Stomatology, Capital Medical University, Beijing, 100050 China

## Abstract

This study aimed to investigate the effects of subpressure on the bond properties of total-etching adhesive to dentin. Thirty-six caries-free premolars were sectioned parallel to the occlusal plane and randomly divided into four groups (n = 9): a control group (C, no treatment) and three subpressure groups, which were treated under 0.8, 0.6 or 0.4 bar after applying adhesives, named S_8_, S_6_ and S_4_, respectively. Afterward, resin was bonded to the dentin surface, and 27 beams (1.0 mm × 1.0 mm) of each group were sectioned. One was selected to observe the bonding interface from each group by SEM. Each group was divided into two subgroups (n = 13): 24 hours of water storage (I) and 10,000 thermocycling (A). The microtensile bond strength (μTBS), failure modes and nanoleakage expression were evaluated. SEM results showed that the subpressure groups had longer and denser resin tags. The μTBS of the subpressure groups was higher than that of the control group (p < 0.05). The subpressure groups were dominated by mixed failure, whereas main interfacial failure appeared in group C. The subpressure groups showed less silver deposition than the control group (p < 0.05). The subpressure technique may remarkably improve bonding strength and decrease nanoleakage on total-etching bonding.

## Introduction

In dental restorative procedures, the dentin bonding system has a great effect on the clinical success. Currently, the bonding system is classified into two parts, the total-etching adhesive system (including 3-step and 2-step systems), and the self-etching system (including 2-step and 1-step systems)^1–3^. Various factors contribute to the bonding properties, such as resin, adhesive, hybrid layers, resin tags, nanoleakage and dentin, in which hybrid layers and resin tags play the key role^4^. To improve the hybrid layers, some researchers have studied the pretreatment of dentin, such as laser etching^5^, sand blasting^6^ and plasma spraying^7^. The others have explored new adhesives to improve the penetration and resin tags length of adhesives^8^. To reach the goal, dentin adhesives have been developed to 7 (and even 8) generations^9^. Compared with classic resin adhesives, one of the improvements of novel adhesives was enhancing the penetration of the adhesives, so that resin tags could become longer. However, the resin penetration process is still incomplete, leaving porosities on the micro- and nano-scale within the hybrid layer that weaken dentin bonding^10^. Nanoleakage was originally described as leakage at nanometer-sized channels, which can occur within the hybrid layer and/or in the adhesive layer. This phenomenon has been widely implicated as an important factor that causes degradation of the bonding to dental tissue^11–13^. Unsatisfactory bonding causes restoration loosening and secondary caries, which wastes much manpower and material resources^14^. Ideal bonding between resin and dentin has not been achieved until now^15, 16^.

To date, the total-etching adhesive system is still considered the gold standard to evaluate the bonding strength^17–22^. The aim of this study was to verify the effectiveness of subpressure technique on dentin-bonding properties using the total-etching adhesive system.

## Results

**Figure 1:**
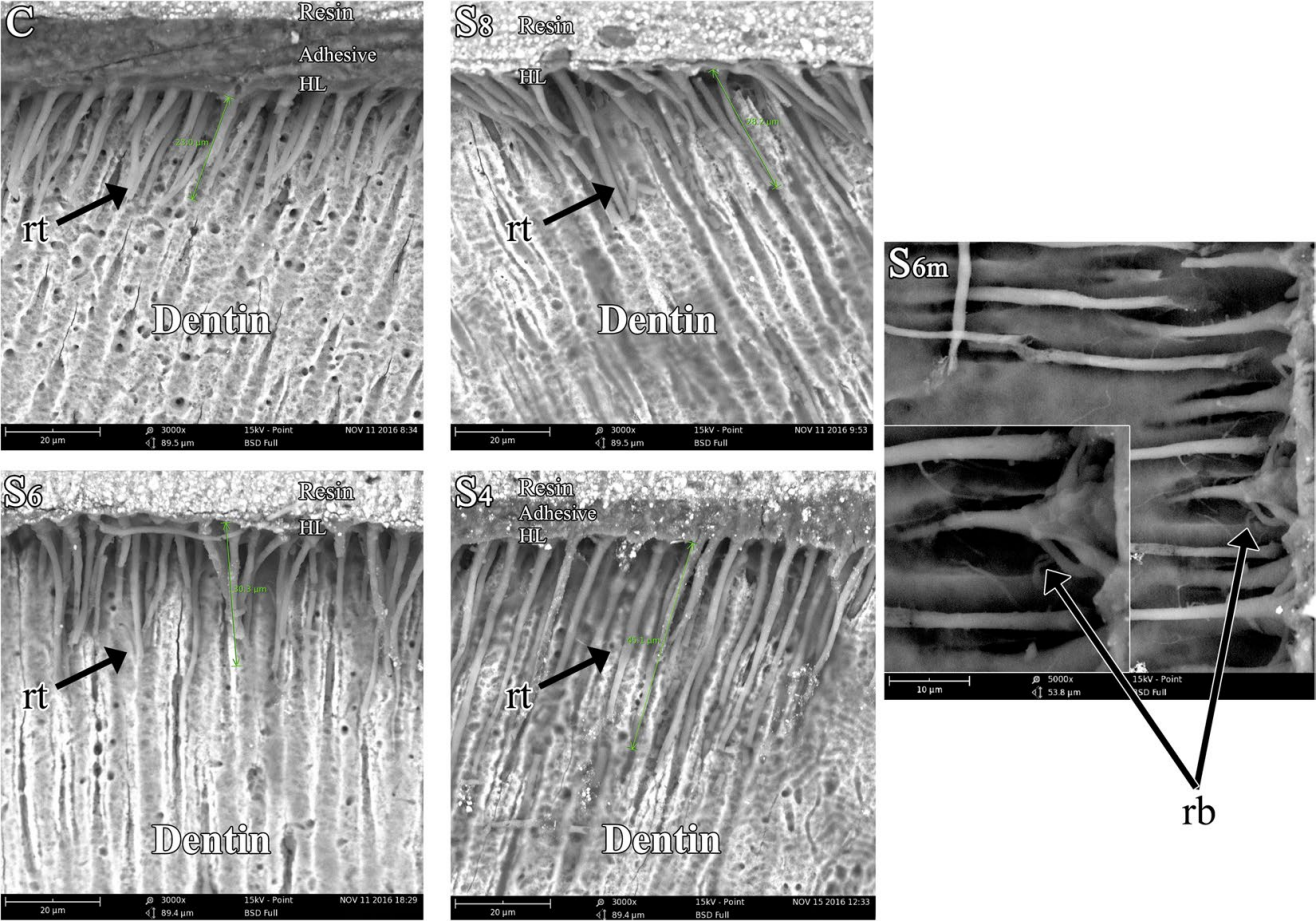
Representative SEM images of the bonding interface. C: Specimen of the control group, with no additional treatment after applying the adhesive and after 24 h of water storage; S_8_: Specimen of group S_8_, in which the dentin surfaces were treated under 0.8 bar after applying adhesives and after 24 h of water storage; S_6_: Specimen of group S_6_, in which the dentin surfaces were treated under 0.6 bar after applying adhesives and after 24 h of water storage; S_4_: Specimen of group S_4_, in which the dentin surfaces were treated under 0.4 bar after applying adhesives and after 24 h of water storage. S_6m_ showed resin tags of S_6_ at high magnifications (×5000, ×10000); (rt) resin tags, (rb) resin branches (nano-resin-tags), (HL) hybrid layer.

SEM observation of the bonding interface after 24 h of water storage is shown in Fig. 1. The intensity of bonding agent tags in group C was obviously sparser than that of the experimental groups. With the decrease in pressure value, the distribution of the resin tags was denser in the experimental groups.

The lengths of the bonding agent tags were measured, and nano-resin-tags are presented in Fig. 1. The length of the resin tags in each group was approximately 23 mm, 28.2 mm, 30.3 mm and 45.2 mm, respectively. The length of the resin tags in the subpressure groups was obviously longer than that of control group. Hybrid layers could be obtained in all groups.

Group S_6_ was selected for analysis at high magnifications (×5000, ×10000), and a nano-resin-tag (rb) was witnessed around the larger tags, which indicated that the dentin tubule branches were filled with the adhesive agent in the experimental groups.

**Figure 2:**
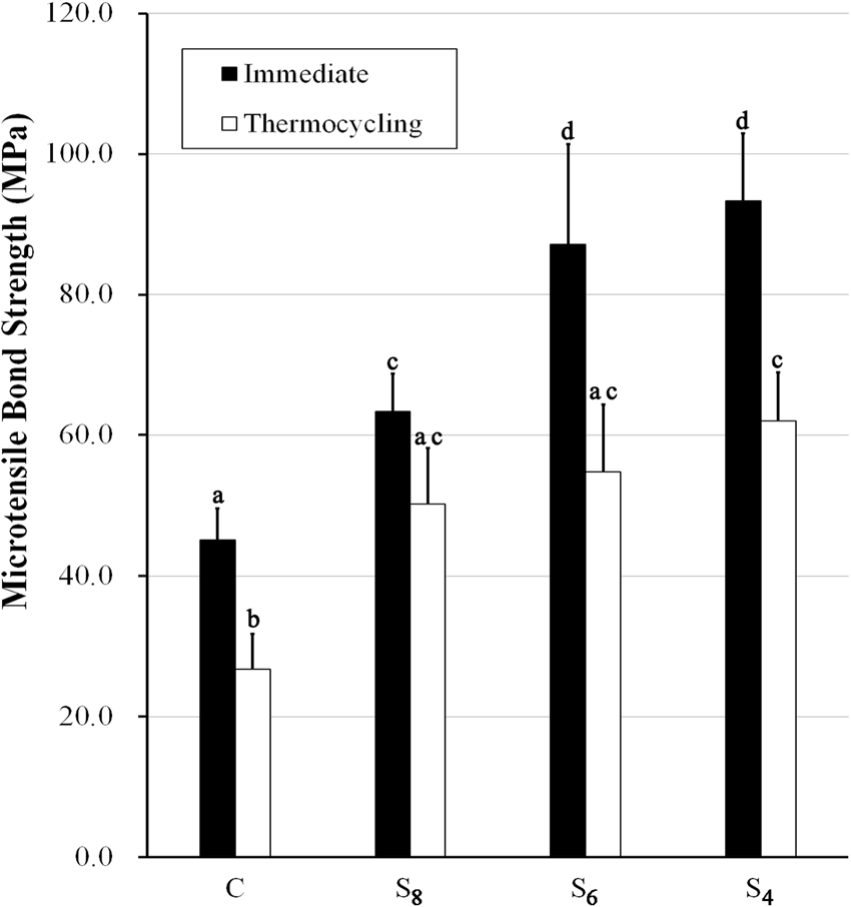
Micro-tensile bond strength of all groups. C: Specimen of the control group, with no additional treatment after applying the adhesive; S_8_: Specimen of group S_8_, in which the dentin surfaces were treated under 0.8 bar after applying adhesives; S_6_: Specimen of group S_6_, in which the dentin surfaces were treated under 0.6 bar after applying adhesives; S_4_: Specimen of group S_4_, in which the dentin surfaces were treated under 0.4 bar after applying adhesives. The black column shows the μTBS of the groups before aging, and the white column shows the μTBS of the groups after aging. Different letters indicate significant difference (p < 0.05).

Figure 2 and Table 1 show the mean μTBS and standard deviation of the groups before and after aging: CI = 45.12 ± 4.46 MPa; CA = 26.75 ± 5.17 MPa; S_8_I = 63.36 ± 5.33 MPa; S_8_A = 50.28 ± 7.85 MPa; S_6_I = 87.16 ± 14.29 MPa; S_6_A = 54.81 ± 9.99 MPa; S_4_I = 93.36 ± 9.59 MPa and S_4_A = 62.04 ± 5.06 MPa, respectively. Two-way ANOVA showed that the subpressure treatment (F = 78.183, P = 0.000) and aging treatment (F = 129.676, P = 0.000) influenced the bond strength significantly. The interaction of subpressure × thermocycling was also significant (F = 5.233, P = 0.003), indicating that the changes in μTBS were dependent on these two factors. Group S_4_I showed the highest bond strength, which was approximately two times than that of group C.

**Table 1:** Statistical analysis of the bonding strength among the groups.

(I) group	(J) group	Mean Difference (I-J)	Std. Error	Sig.	95% Confidence Interval	
					Lower Bound	Upper Bound
CI	CA	18.37375*	2.41350	0.000	9.0804	27.6671
	S_8_I	−18.24000*	2.45882	0.000	−27.7293	−8.7507
	S_8_A	−5.15875	3.19203	0.982	−18.1396	7.8221
	S_6_I	−42.03500*	5.29186	0.001	−65.7770	−18.2930
	S_6_A	−9.69250	3.86707	0.595	−26.1068	6.7218
	S_4_I	−48.23750*	3.73937	0.000	−63.9967	−32.4783
	S_4_A	−16.92000*	2.38466	0.000	−26.0908	−7.7492
CA	S_8_I	−36.61375*	2.62616	0.000	−46.6803	−26.5472
	S_8_A	−23.53250*	3.32264	0.000	−36.7214	−10.3436
	S_6_I	−60.40875*	5.37166	0.000	−84.0147	−36.8028
	S_6_A	−28.06625*	3.97556	0.001	−44.5043	−11.6282
	S_4_I	−66.61125*	3.85146	0.000	−82.4212	−50.8013
	S_4_A	−35.29375*	2.55685	0.000	−45.0934	−25.4941
S_8_I	S_8_A	13.08125	3.35571	0.055	−0.1759	26.3384
	S_6_I	−23.79500*	5.39217	0.047	−47.3747	−0.2153
	S_6_A	8.54750	4.00324	0.805	−7.9104	25.0054
	S_4_I	−29.99750*	3.88002	0.000	−45.8346	−14.1604
	S_4_A	1.32000	2.59968	1.000	−8.6491	11.2891
S_8_A	S_6_I	−36.87625*	5.76357	0.002	−60.4540	−13.2985
	S_6_A	−4.53375	4.49101	1.000	−21.9607	12.8932
	S_4_I	−43.07875*	4.38153	0.000	−60.0171	−26.1404
	S_4_A	−11.76125	3.30176	0.104	−24.9100	1.3875
S_6_I	S_6_A	32.34250*	6.16311	0.005	8.0898	56.5952
	S_4_I	−6.20250	6.08380	1.000	−30.2816	17.8766
	S_4_A	25.11500*	5.35876	0.034	1.4908	48.7392
S_6_A	S_4_I	−38.54500*	4.89520	0.000	−57.3119	−19.7781
	S_4_A	−7.22750	3.95812	0.942	−23.6557	9.2007
S_4_I	S_4_A	31.31750*	3.83346	0.000	15.5220	47.1130

CI = specimens with no additional treatment and after 24 h of water storage; CA = specimens with no additional treatment and after 10,000 thermocycles; S_8_I = specimens with 0.8 bar and after 24 h of water storage; S_8_A = specimens with 0.8 bar and after 10,000 thermocycles; S_6_I = specimens with 0.6 bar and after 24 h of water storage; S_6_A = specimens with 0.6 bar and after 10,000 thermocycles; S_4_I = specimens with 0.4 bar and after 24 h of water storage; S_4_A = specimens with 0.4 bar and after 10,000 thermocycles. *p < 0.05.

**Figure 3:**
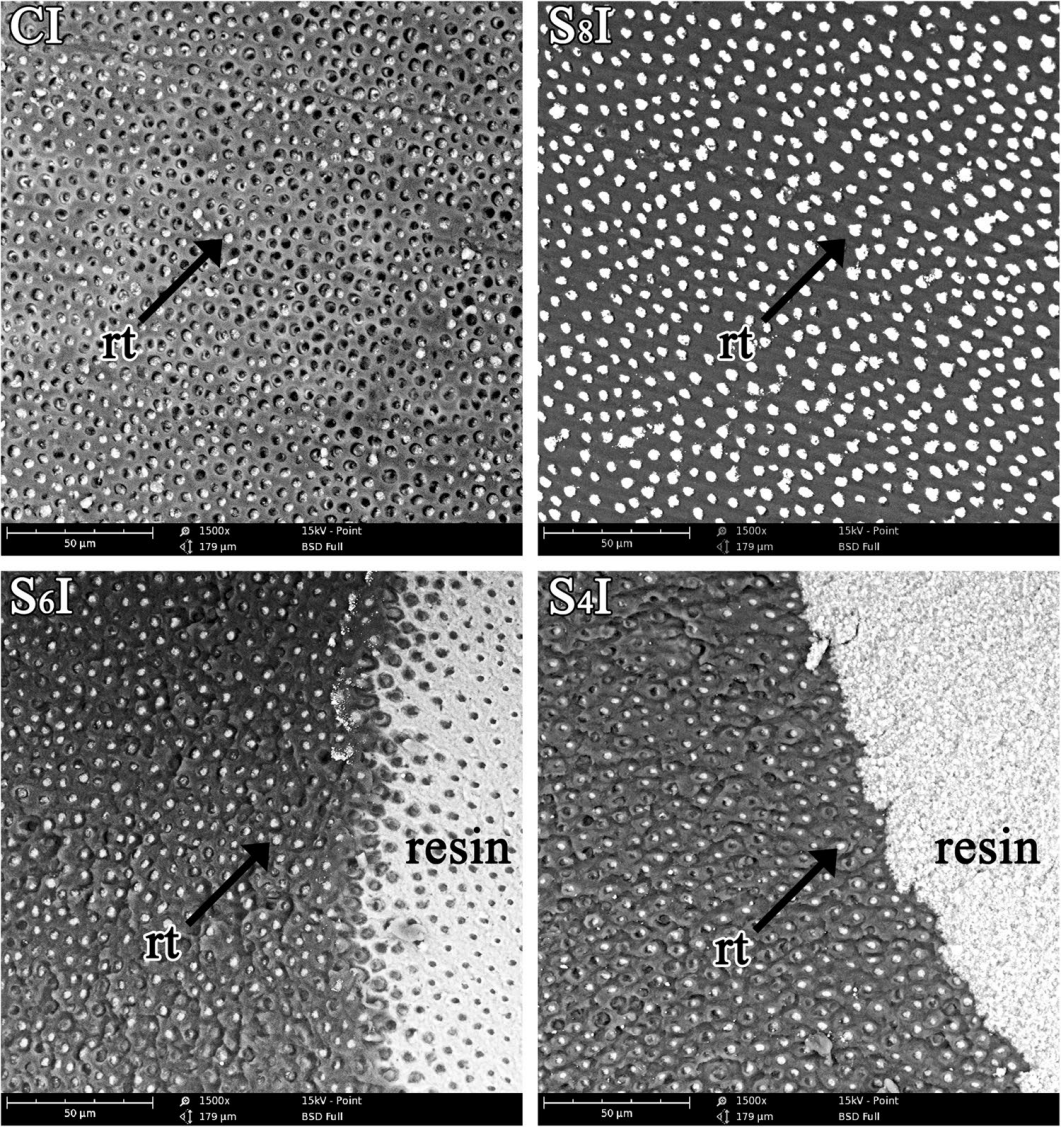
Representative debonding surface of each group. CI: Representative debonding surface of group CI, in which there is less adhesive left on the dentin surface and some resin tags have broken away from the dentinal tubule; S_8_I: Representative debonding surface of group S_8_I, almost all the dentinal tubule is still filled with resin; S_6_I: Representative debonding surface of group S_6_I; not only the resin tags but also resin can be obtained on the debonding dentin surface. S_4_I: Representative debonding surface of group S_4_I, which shows a similar result as S_6_I.

Figure 3 shows the representative fracture interfaces of the dentin side after micro-tensile test. In group CI, the main fracture of the tags was in the dentin tubule. However, resin tags were not found in some of the tubules. In group S_8_I, S_6_I and S_4_I, all dentin tubules were filled with resin tags on exposed dentin. The failed fracture occurred mainly on the interface between dentin and resin in group S_8_I. In group S_6_I and S_4_I, the dentin was exposed in some areas of the failed fracture interface, while resin could be observed in the remaining areas. The distribution of the failure modes is summarized in Table 2.

**Table 2:** Fracture mode, and nanoleakage expression of each group*.

Groups	Failure Mode (%)				Nanoleakage Expression (%)
	IF	CC	CD	M	
CI	62.5	12.5	0	25	7.90 ± 3.17^A^
CA	75	0	0	25	13.02 ± 2.35^B^
S_8_I	25	12.5	12.5	50	4.26 ± 1.95^C^
S_8_A	25	0	12.5	62.5	9.11 ± 3.05^A^
S_6_I	12.5	25	0	62.5	3.70 ± 1.68^C^
S_6_A	12.5	0	12.5	75	7.04 ± 1.78^A^
S_4_I	0	12.5	12.5	75	1.57 ± 1.00^D^
S_4_A	0	12.5	0	87.5	3.21 ± 0.71^C,D^

Abbreviations: IF, interface failure; CC, cohesive failure in the composite; CD, cohesive failure in the dentin; M, mixed failure.

Chi-square analysis revealed a significant difference in the failure mode between different groups (P = 1.3032E-67).

*Nanoleakage values are expressed as the mean ± standard deviation. Groups with the same letters do not significantly differ (p < 0.05).

The failure pattern in groups CI and CA was mainly interface failure in either the dentin or resin composite, while the other groups predominantly exhibited mixed failure. The ratio of mixed fractures in the experimental group was significantly higher than that in group C (p < 0.05). The proportion of the mixed fracture mode was increased significantly with decreased subpressure values (p < 0.05).

**Figure 4:**
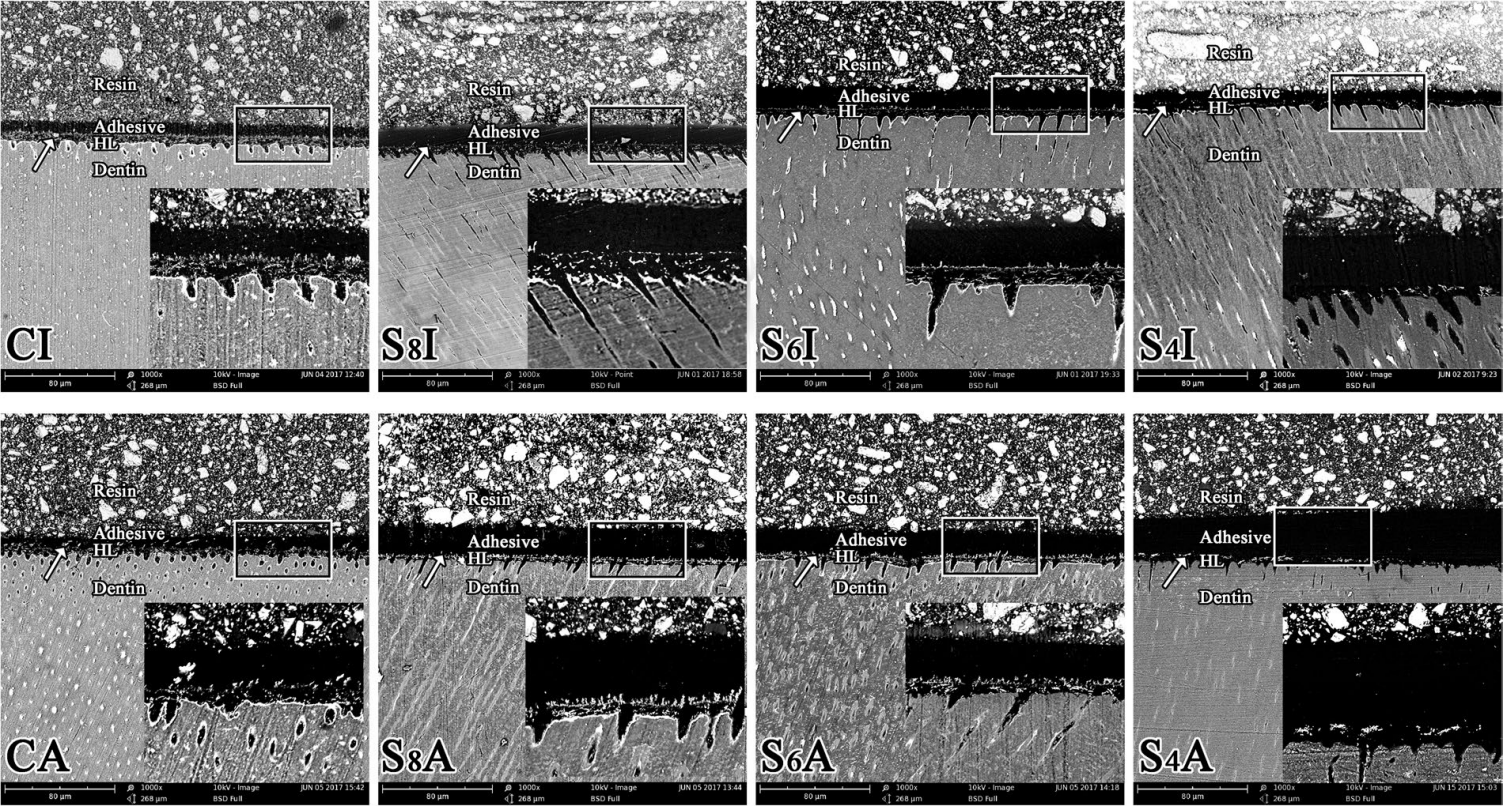
Representative SEM-BSD images (×1000) of the interfacial nanoleakage expression of different groups. HL: hybrid layer. Arrow: silver deposits. CI & CA, control groups; S_8_I, S_8_A, S_6_I, S_6_A, S_4_I and S_4_A, subpressure groups.

Representative images of silver-challenged specimens for eight groups were illustrated in Fig. 4. High magnification SEM images (×1000, ×2500) showed that both the subpressure treated and control beams presented a silver infiltrate into the base of the hybrid layer before and after aging (Fig. 4). Subpressure treatment yielded a lower percentage of silver deposition compared with the control group on the same aging period (p < 0.05). The lower the subpressure applied, the lower the percentage that was obtained. Less silver deposition was observed in group S_6_ and S_4_ after aging than in the control group before the aging treatments (p < 0.05) (Table 2). A distinctive silver-spotted pattern of nanoleakage formation was observed along the adhesive/dentin interface in all specimens, and its silver percentage distribution can be visualized in Fig. 4 and Table 2.

## Discussion

Dentin bonding has been studied for decades. The total-etching system is a proven technology with effective bonding to enamel and dentin both *in vitro* and *in vivo* and is still considered the gold standard for testing new adhesive systems^17–22^. Clinically, the characteristics of the total-etching system, such as the complex operation, highly sensitive technology and dentin hypersensitivity, can easily cause doctors to choose self-etching adhesive systems. The shedding rate of self-etching adhesive systems is 31%, 49% and 60.3% by clinical observation of resin-dentin bonding for 3 years, 5 years and 13 years, respectively^23^, although self-etching adhesive systems can offer a convenient method of bonding and an acceptable initial bond strength. Meanwhile, poor bonding durability may weaken retention, produce marginal deterioration, and reduce the service life of restorations^4^. Therefore, novel technologies have been extensively investigated in dentistry to improve the dentin bonding durability.

In this study, the subpressure technique applied to the bonding surface was as follows: The dentin tubule was sealed when the dentin surface was treated with the adhesive agent. The gas in the dentin tubules was exhausted when subpressure was held on the dentin surface, and the inner and outer pressures of the tubule were balanced, and the adhesive agent flew and sealed the dentin tubule again. When the subpressure was released, the atmosphere automatically pressed the bonding agents into a deeper part of the dentin tubule. Thus, the bonding agent could penetrate into the deep portion of the dentin and more closely combine with the dentin. Moreover, the vacuum area is usually larger than the preparation area, and the dimensions of the vacuum chuck must be adequate to satisfy the need for convenient sealing.

As shown in the SEM images (Fig. 1), the subpressure technique effectively enhanced the penetration of resin monomer into dentin tubule. Compared with the control group, the subpressure groups had longer resin tags and a denser distribution because the subpressure technology ejected the bonding agents deeply into the dental tubule. In addition, the representative SEM images indicated that resin tags were well-infiltrated into the dentinal tubules, and even the dentin tubule branches could be filled by nanoscale tags. These made the adhesive and dentin issue combined more tightly, which was helpful to improve the bonding property^24^. Therefore, the micro- and nano-scale porosities within the hybrid layer could be reduced, and the bonding retention could be increased^10^.

μTBS results indicated that the bonding strength values were increased with the decrease of the pressure value. The subpressure technique could improve the bonding strength. With the decrease in the pressure value, the bonding strength was nonlinearly increased. Group S_4_I reached the highest bond strength (93.36 ± 9.59 MPa), which was approximately two times higher than group CI because the resin tags became longer and combined with the dentin closely under the highest subpressure. Figure 2 also shows that the bonding strength values of the subpressure groups after aging were still higher than that of group CI. Notably, the subpressure application, particularly in the experimental group, improved not only the immediate adhesive–dentin bonding but also the bonding stability after aging. This phenomenon was considered the result of subpressure displaying superior performance and eliciting a synergistic effect on the dentin bonding.

As shown in Fig. 3, the representative fracture interfaces showed some severed tags in the dentin tubules in group CI, resulting in a low bonding strength between the adhesive agent and dentin. The ratio of mixed fractures was obviously positively correlated in intensity with an increase in the subpressure. The lowest pressure (0.4 bar) gained the highest bonding strength and the maximum ratio of mixed fracture, which was consistent with previous studies^25^. The subpressure amplified the penetration of the bonding agent to dentin. The dentin interface hybrid layer and resin were mixed together to form the mechanical locking function^14, 26^. In this study, the subpressure technique improved the hybrid layer and resin tags. In group S_8_I, S_6_I and S_4_I, all the dentin tubules were filled with resin on the exposed dentin surface, and the cohesive failure trend grew with the increasing subpressure (Fig. 3). Therefore, the subpressure technique provided a high retention force of bonding resin restorations.

By contrast, the subpressure technique caused the dentin to closely combine with the adhesive agents, which may protect collagen fibers from physical and chemical degradation and improve the stability of dentin bonding^27^.

Nanoleakage was originally used to describe the microporous zone beneath or within hybrid layers that permit tracer penetration to occur in the absence of interfacial gaps^28^. It occurs through submicrometer-sized spaces within dentin hybrid layers where disparities exist between the depths of demineralization and monomer diffusion^29^. However, the hybrid layer is inevitable in dentin bonding. Due to the existence of nanoleakage, the unprotected collagen fiber may be vulnerable to degradation by oral and bacterial enzymes, thus reducing the service life of resin^30, 31^. It may be caused by insufficient infiltration of resin into the demineralized collagen network or by incomplete polymerization of hydrophilic monomers in the submicron interfacial spaces. The results of nanoleakage evaluation revealed less silver deposition in the subpressure groups than in the control group. The subpressure-treated specimens barely had silver deposition in the bonding interface before and after aging (Fig. 4). This result demonstrates that subpressure can effectively prevent water from intruding into the dentin-resin bond interface, decrease water leakage on the interface, and stabilize the dentin bond strength. These phenomena can be explained by the gas in the collagen fiber being released during subpressure treatment in a vacuum. Then, the adhesive agent more closely combines with the collagen fiber as the vacuum is released.

Clinically, the vacuum chuck can be easily accessed to a dental chair just like suction. Otherwise, the vacuum chuck can also be accessed to a mini-vacuum pump for clinical use. Therefore, the subpressure technique might have a broad clinical application.

Within the limitations of this study, subpressure offered a reliable interfacial morphology, improved the short- and long-term bonding strength, and reduced nanoleakage, which showed a positive effect on the total-etching dentin bonding and increased bond durability. However, the most appropriate subpressure value and other bonding systems must be reported in the near future.

## Materials and Methods

### Subpressure apparatus

A homemade apparatus was used to provide subpressure in this study, which consists of four parts, a vacuum chuck, a hollow handle, a governor valve and a vacuum pump.

- The vacuum chuck had a rubber edge and provided a seal between the vacuum chuck and teeth. The dimensions of the vacuum chuck must be adequate to satisfy the need for sealing.
- The hollow handle had a three-way valve, which could switch the passageway to the pump or outside.
- The governor valve included a vacuum gauge and a vacuum relief valve. The vacuum relief valve could adjust the relative vacuum degree, and the degree could be obtained by the vacuum gauge.
- The vacuum pump was used to provide the vacuum.

**Figure 5:**
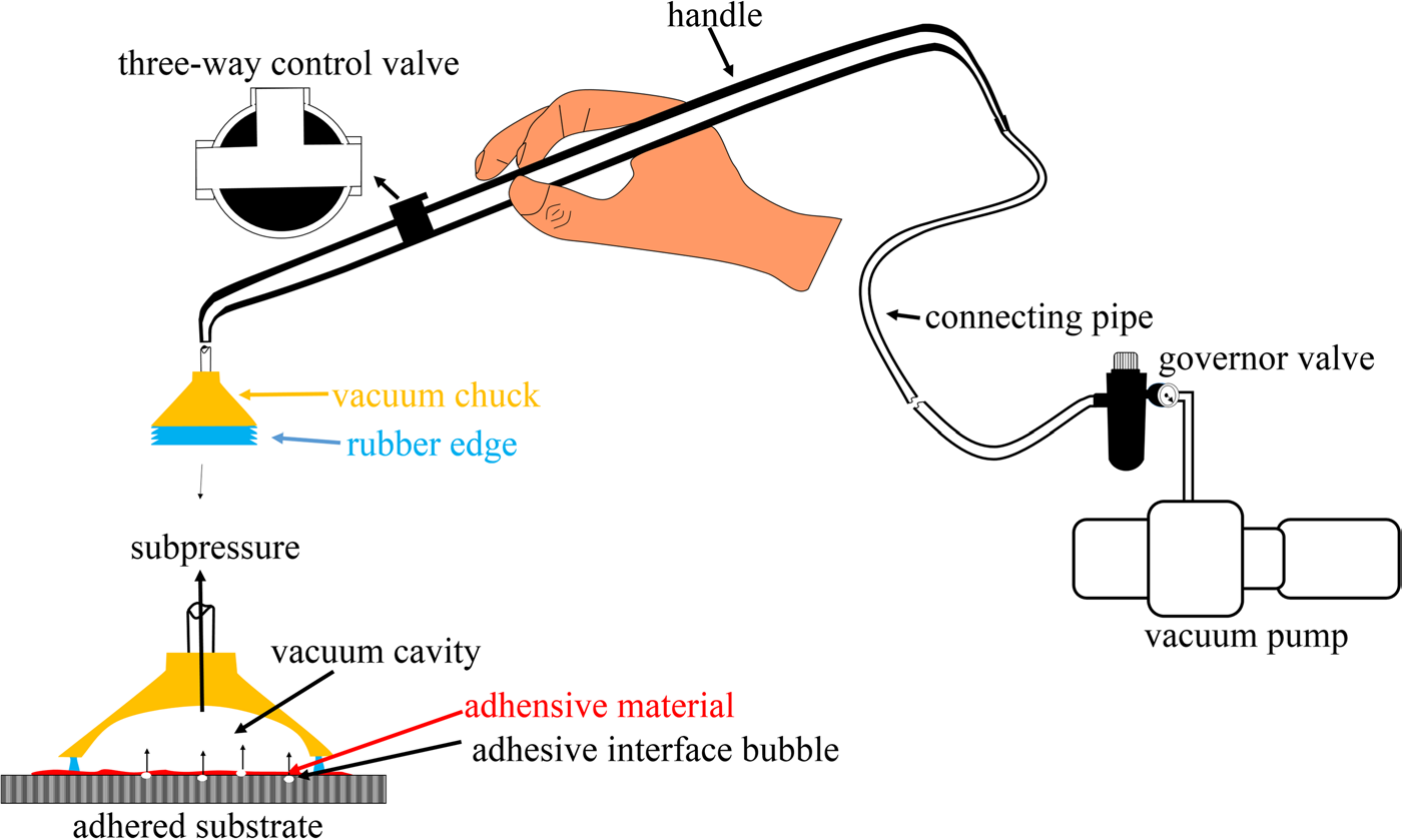
Apparatus of the subpressure technique.

The hollow handle and governor valve were connected by a connecting pipe. The edge of the vacuum chuck and the adhered substrate closely contacted each other under the subpressure condition. When the control valve was switched to the pump side and opened the pump, the relative vacuum degree in the vacuum chuck could be adjusted by the vacuum relief valve. The subpressure could be released when the control valve was switched to the outside. The schema of the homemade apparatus is shown in Fig. 5.

### Specimen preparation

The studies were conducted in accordance with the protocol approved by the Ethics Committee for Human Studies of the Capital Medical University. Thirty-six caries-free human premolars were collected from the Department of Oral and Maxillofacial Surgery of Beijing Stomatological Hospital Affiliated Capital Medical University, after obtaining the patients’ informed consent; the premolars were freshly extracted and stored in artificial saliva at 4 °C for a maximum of 30 days. All the teeth were embedded in an acrylic mold with methylmethacrylate resin. The occlusion enamel of the teeth was removed with 600-grit SiC paper with continuous water cooling in a polishing machine (YUZHOU, China). After ultrasonic cleaning in distilled water for 5 min, these teeth were randomly divided into a control group (C, no treatment) and three subpressure groups (S), with nine teeth in each.

The total-etching dentin-bonding system (3 M Single Bond 2) was utilized in this experiment. All teeth were treated with 35% phosphoric acid for 15 seconds. The control group (C) was handled according to the manufacturer’s instructions. For the subpressure groups (S), the vacuum chuck of the homemade subpressure apparatus was tightly placed on the tooth surface after the adhesive agent was applied. According to the different pressures of 0.8, 0.6 and 0.4 bar on the adhesive surface produced by the apparatus, the experimental groups were named as S_8_, S_6_ and S_4_, respectively. The subpressure was maintained for 10 s, released for 15 s, and then the adhesive surface was blown and cured (Elipar^TM^ 2500, 3 M ESPE, USA). The column-shaped resin composites (Valux^TM^ Plus, 3 M ESPE, USA) that were accumulated over-covered the dentin surface with a height of approximately 5 mm. The specimens were left at room temperature for over 30 min to ensure the initial polymerization.

After the teeth were stored in deionized water at 37 °C for 24 h^32^, the bonded teeth were longitudinally sectioned under a water-cooled diamond saw (Isomet 4000 Linear Precision Saw, Buehler, USA) to produce slabs with a thickness of 1.0 mm. Three slabs from each tooth were sectioned again to prepare beams with a dimension of 1.0 mm × 1.0 mm. After excluding unqualified beams, which were situated peripherally and showed the presence of enamel, twenty-seven beams with a complete bonding interface were selected randomly from each group and kept in distilled water at 37 °C.

### Bonding interface observation

One beam (after 24 h of water storage) was randomly selected from each group to observe the bonding interface. Selected specimen surfaces were etched with 15% phosphoric acid for 10 secs to bring the polished surfaces into relief. Then, these specimens were mounted on aluminum stubs and examined by SEM (Phenom-world Co., Ltd., Netherlands).

### Artificial Aging Treatment

The left beams with each surface treatment were further divided into the following two subgroups (n = 13):

Group I (immediate): Beams without any artificial aging treatment served as the baseline, named CI, S_8_I, S_6_I, and S_4_I, respectively.

Group A (thermocycling): Beams perform a thermocycling test from 5 °C to 55 °C for 10,000 cycles at a dwell time of 30 seconds per temperature and a transfer time of 10 seconds between baths (Temperature Cycling Chambers; TC-501F, WELL, Suzhou, China)^33^, named CA, S_8_A, S_6_A, S_4_A, respectively.

### Micro-tensile bond strength test

Eight beams of each group were subjected to micro-tensile bond strength (μTBS) testing. The μTBS was tested on the fixed apparatus at a crosshead speed of 0.5 mm/min by the micro tensile tester (Micro Tensile Tester T-61010K, Biscotto, USA) according to ISO standards^34^ The bonding area was calculated by measuring the size of the bonding interface with a vernier caliper (Wuxi Tin Blade Cutter Co. Ltd., China). The μTBS value was calculated according to the following formula:

$${\rm{\mu }}\text{TBS}={\rm{F}}/{\rm{S}}$$

where μTBS is the micro-tensile bond strength (MPa), F is the maximum load debonding force (N) and S is bonded area (mm^2^).

### Fracture surface analysis

The dentin side of the bonding interface was observed after the debonding. The failure mode of each specimen was inspected with SEM and classified into three categories, as the follows:

1. Cohesive failure, including (a) the cohesive failure of the dentin in dentin and (b) the cohesive failure of the resin and/or adhesive in the adhesive layer and/or the resin layer.
2. Interface failure, between the surface of dentin and adhesive layer, with very little adhesive on the dentin side (except the resin tags of the adhesive resin) and scarcely any dentin on the resin side.
3. Mixed failure, in which the dentin of the tooth has a large amount of bonding agent and resin, and the resin side retains a large amount of dentin.

### Nanoleakage Evaluation

Five beams of each group were subjected to a nanoleakage evaluation using the method described by Tay and others^30, 31^. Each beam was coated with two layers of nail varnish applied 1 mm from the bonded interface, followed by immersion into 50 wt% ammoniacal AgNO_3_ in darkness for 24 hours, thoroughly rinsed in distilled water, and placed in a photodeveloping solution for 8 hours under a fluorescent light. Each beam was then wet-polished until 2500# SiC paper and ultrasonically cleaned and dehydrated for 24 hours at room temperature. Interfacial images were obtained by SEM (BSD mode) for four random areas of each beam at ×1000 magnification for quantitative analysis. The percentage distribution of metallic silver particles within the adhesive-dentin interfaces were calculated with Photoshop (Adobe Photoshop CC2017, Adobe Systems Software Ireland Ltd, USA) using a threshold tool.

**Figure 6:**
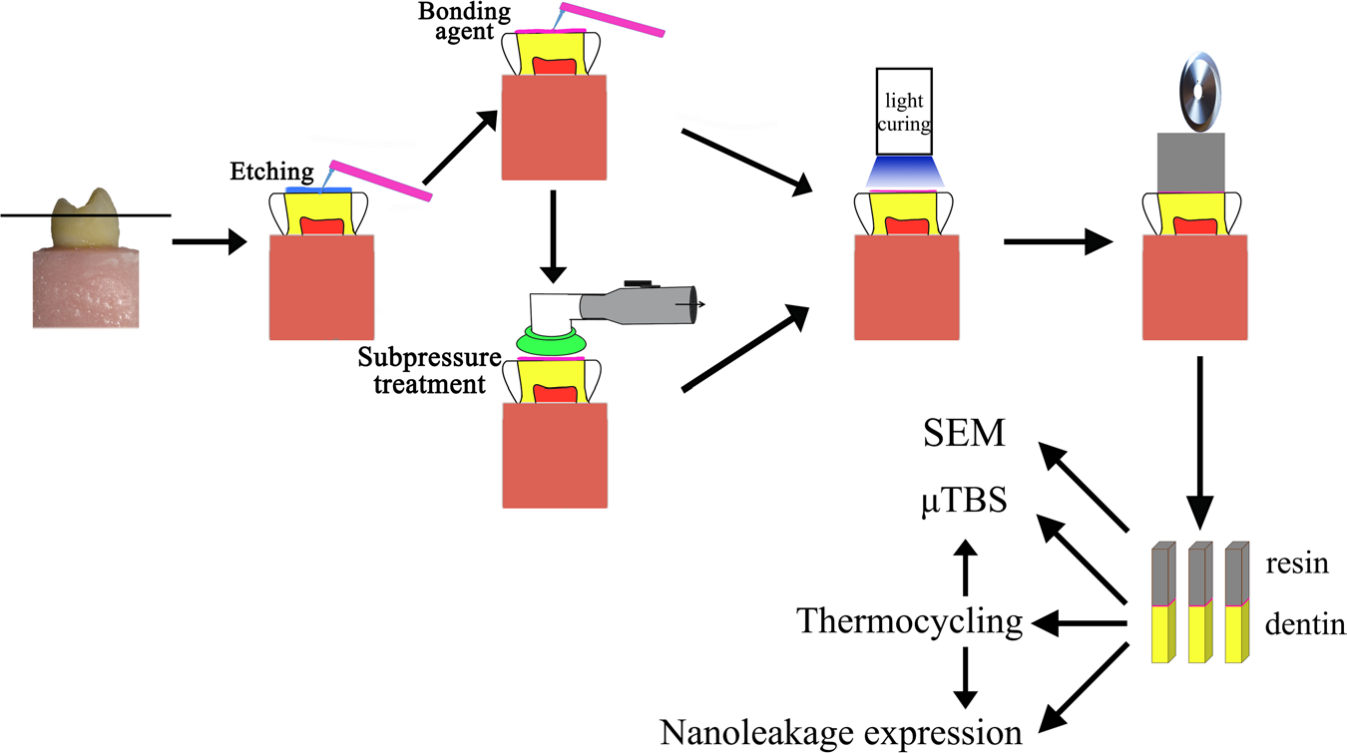
Schematic diagram of the specimen preparation.

The schematic diagram of the preparation of specimens is shown in Fig. 6, and these preparations conform to the ISO standard^34^.

### Statistical analysis

The data are presented as the mean ± SD. The µTBS value was analyzed using two-way ANOVA factorial analysis followed by Tukey’s post hoc multiple comparison test with SPSS (SPSS 22.0, IBM for Windows), to analyze the effect of two variables (subpressure and thermocycling) on bond strength and nanoleakage expression. The failure mode was statistically analyzed using the Pearson Chi-square test with the same software. A value of p < 0.05 was considered statistically significant.
